# Gliomatosis cerebri type II: two case reports

**DOI:** 10.4076/1752-1947-3-7225

**Published:** 2009-06-15

**Authors:** Pietro Ivo D’Urso, Oscar Fernando D’Urso, Santo Marsigliante, Carlo Storelli, Alessandro Distante, Francesca Sanguedolce, Antonia Cimmino, Giuseppe Luzi, Cosimo Damiano Gianfreda, Antonio Montinaro, Pasqualino Ciappetta

**Affiliations:** 1 Department of Neurosciences - Section of Neurosurgery, University of Bari Medical School Bari Italy; 2 Department of Biological and Environmental Sciences and Technologies (DiSTeBA) Ecotekne, Via Prov.le per Monteroni, 73100 Lecce Italy; 3 CNR-IFC Lecce section Ecotekne, Via Prov.le per Monteroni, 73100 Lecce Italy; 4 Department of Pathology - Section of Neuropathology, University of Bari Medical School Bari Italy; 5 Department of Clinical Immunology and Allergology, Sant’Anrea Hospital Rome Italy; 6 Neurosurgery Operative Unit, V. Fazzi Hospital 73100 Lecce Italy

## Abstract

**Introduction:**

Two types of gliomatosis cerebri exist: Type I and Type II. We report the results of a histological and genetic study of two cases of gliomatosis cerebri Type II, correlating these results with therapy and prognosis.

**Case presentation:**

Two patients, a 52-year-old man (Patient 1) and a 76-year-old man (Patient 2) with gliomatosis cerebri II were admitted to our institution; they underwent surgical treatment and received radiotherapy and chemotherapy. At the 24-month follow-up, Patient 1 was still alive, while Patient 2 had died. The poor prognosis of Patient 2 was underlined by molecular analysis which showed that the angiogenesis related genes *VCAM1* and *VEGF* were overexpressed, reflecting the high degree of neovascularization.

**Conclusion:**

Genes involved in drug resistance and metallothioneins were highly expressed in Patient 2 and this, associated with unmethylated O6-methylguanine methyltransferase, can explain the lack of response to chemotherapy.

## Introduction

Gliomatosis cerebri (GC) is a diffuse, frequently bilateral, glial tumor which infiltrates the brain, involving more than two lobes. It often extends to the infratentorial structures and even to the spinal cord. According to the current WHO classification of brain tumors, GC is a distinct malignant neuroepithelial neoplasm of uncertain origin [1]. Pathologists describe two types of GC: Type I is the classic form of GC characterized by diffuse overgrowth with neoplastic glial elements without a focal mass presence. Type II may stem from Type I and is characterized by a diffuse brain infiltration and focal mass presence, usually a high-grade glioma [1]. More than 200 cases of GC have been described in the literature, but very few cases of Type II GC have been genetically typed. We report the results of a histological and genetic study in two cases of gliomatosis cerebri Type II. A prognostic correlation of these results is also provided.

## Case presentation

Two patients of Italian ethnicity, a 52-year-old man (Patient 1) and a 76-year-old man (Patient 2), with clinical signs of increased intracranial pressure were admitted to our institution. Pre-operative neuroradiological studies (computed tomography (CT) scan and magnetic resonance imaging (MRI)) were performed. The patients underwent surgical treatment and received radiotherapy at a 54.9 Gy dose and concomitant chemotherapy with temozolomide.

Fresh surgical specimens were frozen in liquid nitrogen immediately after removal, and were treated immediately upon arrival at the laboratory. Isolation of DNA from these specimens was performed using the ChargeSwitch gDNA Micro Tissue Kit (Invitrogen, Karlsruhe, Germany). Isolation of total RNA from the surgical specimens was performed using the PureLink™ Micro-toMidi™ Total RNA Purification System following the manufacturer’s instructions (Invitrogen).

*TP53* exons 5-8 and all *PTEN* exons were analyzed by the sequencing of various tumor regions. Each exon was polymerase chain reaction (PCR) amplified. Sequence reactions were performed with a BigDye Terminator v3.1-kit (Applied Biosystems, Warrington, UK) using both forward and reverse primers. Finally, the sequences were read by an automated sequence reader (ABI PRISM 3130, Applied Biosystems).

For CGH analysis, a custom 60-mer amino modified oligo-microarray was used, containing 6000 spots, specific to 287 chromosomal regions implicated in cancer. Labeling reactions were performed on purified restricted DNA using the BioPrime Array CGH Genomic Labeling System (Invitrogen). Experimental and reference targets were hybridized for 14 to 18 hours at 42°C. After washing, slides were scanned using an Affymetrix 428 array scanner (Affymetrix, Santa Clara, CA, USA).

For LOH analysis, a custom 30-mer amino modified oligo-micro array was used. Single nucleotide polymorphisms (SNPs) were chosen to represent markers for 2q14.3, 2q22.1, 3q13, 7q22, 10q22-10q23, 12q14, 13q14, 16q22 17p.13.1, and 19q13.2. Two 30-mer oligonucleotides per locus were designed to represent the alternate alleles of each SNP. Each oligonucleotide consisted of a 12-nucleotide hairpin and an 18- to 21-nucleotide target sequence with the polymorphic base in the middle.

A custom 60-mer amino modified oligo-array was used, containing 800 probes specific for genes commonly altered in cancer. RNA was labelled using the SuperScript Indirect RNA Amplification System (Invitrogen). The resulting amplified amino-allyl aRNA was dyed using Cy3 for test samples and Cy5 for reference samples.

The microarray expression results were validated by quantitative real-time PCR, using an ABI 7500 Real-Time PCR System (Applied Biosystems). The Wilcoxon signed-rank test was used to analyze statistical significance.

The DNA methylation patterns in the CpG island of the O6-methylguanine-DNA methyltransferase (MGMT), p16, deleted in colorectal cancer (DCC) and death-associated protein kinase 1 (DAPK1) genes were determined by sodium bisulfite treatment and subsequent methylation-specific PCR MS PCR. Primers for MS PCR were designed to encompass the CpG-rich area of the promoter region around the transcription initiation site.

The neuroradiological studies (CT scan and MRI) indicated gliomatosis cerebri Type II. Both patients underwent surgical removal of the focal mass and biopsies in the adjacent areas, and received temozolomide and radiation therapy postoperatively. At the 24-month follow-up, after chemo- and radiation therapy, Patient 1 was still alive with impaired neurological conditions, while Patient 2 had died.

**Figure 1.**
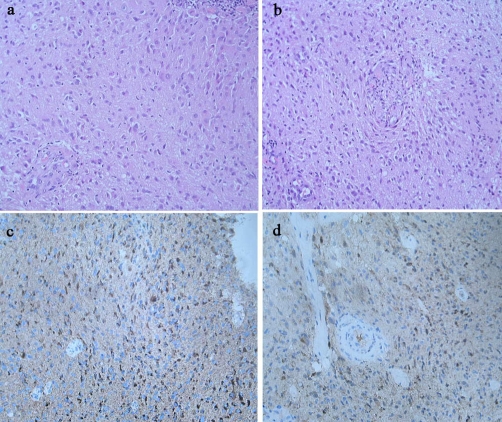
Histological study of the removed masses revealed a diffuse proliferation of neoplastic spindle cells with rod-shaped nuclei infiltrating the brain parenchyma in both Patients 1 (**a**) and 2 (**b**). Paraffin-embedded sections were stained by the immunoperoxidase method and were positive for glial fibrillary acidic protein (GFAP) (**c**) and S100 protein (**d**).

The histological study on the removed masses revealed a diffuse proliferation of neoplastic spindle cells with rod-shaped nuclei infiltrating the brain parenchyma in both patients. Such cells appeared arranged in short chains parallel to nervous fibers, in the shape of a high-grade malignant neoplasm. These aspects were compatible with the pre-operative diagnosis of gliomatosis cerebri Type II. Paraffin-embedded sections were stained by the immunoperoxidase method which yielded the following results: positive staining reaction to glial fibrillary acidic protein (GFAP) and to S100 protein (Figure 1).

**Figure 2.**
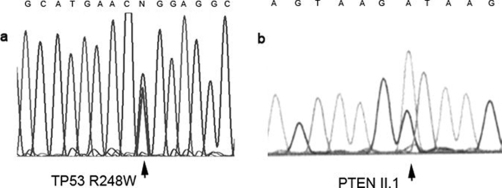
(**a**) Sequence around codon 248 of TP53 showing the mutated base which determines an arginine to tryptophan substitution. (**b**) The PTEN point mutation at the intron 2 splice donor site causes a defective splicing which likely results in a non-functional protein.

In Patient 1, bidirectional sequencing revealed a mutation in codon 248 of *TP53*, with arginine to tryptophan substitution (Figure 2a). Patient 2 carried an intronic point mutation in the *PTEN* gene, which altered the splice donor site within intron 2 and therefore likely caused expression of an aberrantly spliced mRNA (Figure 2b). These same mutations were observed in all of the tumor regions.

CGH analysis did not detect any *CDKN2A* (9p21) deletion (confirmed by LOH) and no *CDK4* or MDM2 amplification in either patient. Patient 2 showed a gain in the *EGFR* gene copy number. These analyses revealed the deletion of the p27kip1 gene locus, no aberrations of *CDKN1A* (p21), and losses of 2q, 3p, 16q and 19q and gains of 7q in both patients (data not shown).

**Figure 3.**
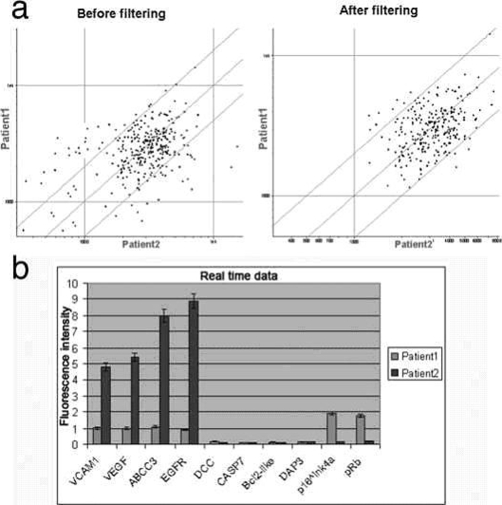
(**a**) Scatterplot showing the gene expression difference between the two patients. Threshold lines are set to a 2-fold change. The left panel shows the data before background filtering, while the right panel shows the data after filtering. (**b**) Histogram showing expression data regarding the selected genes by real-time RT-PCR. Normalization was conducted on beta-actin.

The results of cDNA gene expression profiling are reported in Table 1, while Figure 3a shows a scatterplot with a 2-fold threshold. Genes found to be overexpressed were:

- transcriptional regulators (including *ZNF258*, *EYA2*, *EGR1*, *JUNB*) and intracellular signal transduction genes (*RGS7*, *EHD3*, *CS1*, *ITPK1*, *GRB2*, *STK2*) in both tumors;
- angiogenesis related genes (*VCAM1*, *VEGF*) and drug resistance genes (*ABCC3, MTIL*) in Patient 2.

**Table 1.** Microarray normalized data

Name	Description	I/C* Patient 1	I/C* Patient 2
*Under-expressed in Patient 2*			
IQGAP	IQ motif containing GTPase-activating protein 1	1.13	0.02
ITPK1	Inositol 1,3,4-triphosphate 5/6 kinase	1.5	1.6
P1M1	Pim-1 proto-oncogene gene	0.99	0.06
PSHL	Phosphoserine phosphatase-like	1.0	0.12
PTGER4	Prostaglandin E receptor 4 (subtype EP4)	0.18	0.16
RDCl	G protein-coupled receptor	1.0	0.11
RGS16	Regulator of G-protein signaling 16	1.12	0.06
CAPG	Capping protein (actin filament), gelsolin-like	1.1	0.2
MSN	Moesin	1.0	0.1
PFN1	Profilin 1	0.98	0.12
PLEK	Plekstrin	1.12	0.11
VIM	Vimentin	1.16	0.03
*Over-expressed in Patient 2*			
VCAM1	Vascular cell adhesion molecule 1	1.01	4.8
VEGF	Vascular endothelial growth factor	1.0	5.4
ABCC3	ATP-binding cassette C (CFTR/MRP)	1.09	8.0
MTIL	Metallothionein 1L	1.1	9.9
EGFR	Epidermal growth factor receptor	0.95	8.9
*Over-expressed in Patient 1*			
p16^ink4a	p16^ink4a	1.92	0.18
pRb	pRb	1.8	0.21
*Under-expressed in both patients*			
DCC	Deleted in colorectal cancer	0.2	0.1
CASP7	Caspase 7, apoptosis-related cysteine protease	0.12	0.12
Bcl2-like	2 (Bcl-w)	0.15	0.14
DAP3	Death-associated protein 3	0.16	0.18
*Over-expressed in both patients*			
EGR1	Early growth response 1	3.1	3.3
EYA2	Eyes absent (Drosophila) homolog 2	4.1	3.6
JUNB	Jun B proto-oncogene	2.9	3.2
ZNF258	Zinc finger protein 258	5.0	6.1
RGS7	Regulator of G-protein signaling 7	15	17.2
EHD3	EH domain containing 3	12.1	13.3
CS-1	Calcineurin-binding protein calsarcin-1	9.8	10.2
ITPK1	Inositol 1,3,4-triphosphate 5/6 kinase	6.1	5.8
GRB2	Growth factor receptor-bound protein 2	3.1	3.4
STK2	Serine/threonine kinase 2	2.9	2.6

*I/C represents the normalized ratio between test channel and control channel.

Genes found to be down-regulated were:

- signal transduction genes (*PSHL*, *P1M1*, *IQGAP*, *RDC1*, *RGS16*) and cytoskeleton related genes (*PFN1*, *MSN*, *PLEK*, *VIM*, *CAPG*) in Patient 2;
- apoptosis genes (*CASP7*, *BCLW*, *DAP3* and DCC in line with methylation status) in both tumors.

Interestingly, p16 and pRb were expressed in Patient 1 and down-regulated in Patient 2, while EGFR was overexpressed only in Patient 2.

### QRT-PCR

Statistically significant changes in gene expression at the *P* = 0.025 level were seen with all transcripts analyzed. The results of real-time analysis are reported in Figure 3b.

### Methylation analysis of MGMT, p16, DCC, DAPK1

**Figure 4.**
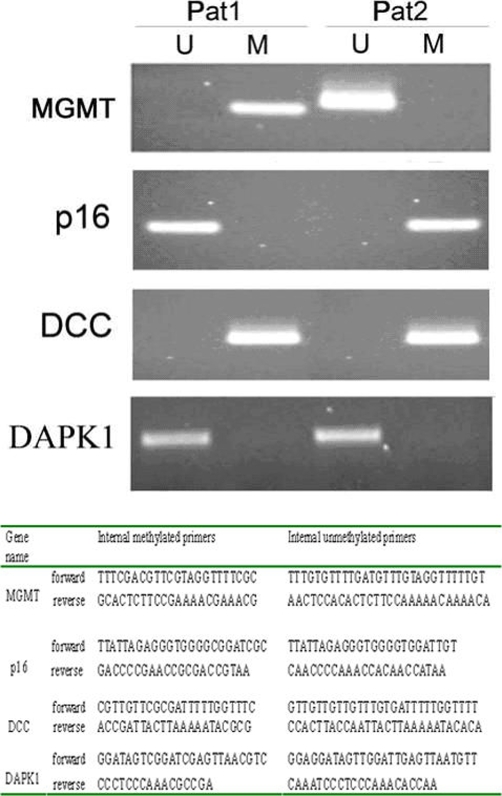
(**a**) Agarose gel electrophoresis relative to MSP products. Lines marked “U” contain products derived from unmethylated DNA templates, whereas products amplified from methylated templates are found in lines “M”. (**b**) Primer sequences used in the methylation-specific PCR to study the methylation status of the promoter of the following genes: MGMT, p16, DCC and DAPK.

DCC was found methylated in both tumors, MGMT methylated only in Patient 1, in line with the chemotherapy response, and p16 methylated only in Patient 2 since this gene is under-expressed even if not mutated (Figure 4a). Primer sequences used in methylation-specific PCR (MSP-PCR) are reported in Figure 4b.

## Discussion

Unlike other cerebral gliomas, gliomatosis cerebri is a particularly invasive glioma subform, characterized by extraordinary brain spreading [2]. Like *TP53* mutations, molecular alterations in gliomatosis cerebri resemble those detected in diffuse astrocytic gliomas [3]. Herrlinger *et al.* described alterations not only in the *TP53* gene, but also in *PTEN* [2]. Our data seem to indicate a mutually exclusive occurrence of both *TP53* and *PTEN* mutations; in fact, Patient 1 had a mutation of *TP53* without any *PTEN* alterations and Patient 2 exhibited a *PTEN* mutation with an intact *TP53*. Genetic aberrations lack cell cycle regulatory genes (*CDKN2A, CDK4*) and the strong expression of p16INK4a and pRb suggests that the pRb-dependent cell cycle checkpoint was intact in Patient 1. Patient 2 showed no genomic aberrations on the *CDKN2A* locus, but had a reduced p16 expression; thus, to understand the mechanism underlying its reduced expression, we investigated the status of its promoter methylation and found it to be hypermethylated: the hypermethylated p16 promoter inhibited its expression [4-7]. In Patient 2, an increase was found in the *EGFR* copy number with its strong expression and *PTEN* mutation; these are all aberrations found in de novo glioblastoma. Interestingly, we found a deletion of the p27kip1 gene locus in both patients, underlining the loss of cell-cycle control. No aberrations were found in *CDKN1A* (p21). We found altered expressions of several genes involved in different cellular process such as those involved in the transcriptional regulation of growth control, and those involved in signal transduction cascades and cytoskeleton related genes. Two angiogenesis related genes, well-known in glioma biology, *VCAM1* and *VEGF*, were overexpressed in Patient 2 reflecting the high degree of neovascularization. Genes involved in drug resistance, *ABCC3* and metallothioneins, which could play a role in drug resistance, were highly expressed in Patient 2’s tumor tissue, explaining his non-responsiveness to chemotherapy. One hallmark of tumors is the lack of control of apoptosis linked genes (*CASP7*, *BCLW*, *DAP3*), found to be down-regulated in both tumors. The absence of *DCC* function, associated with several types of tumors, appeared to also affect our patients. Moreover, we investigated the methylation status of the DNA repair protein gene O6-methylguanine-methyltransferase (MGMT), which is a marker of resistance to chemotherapeutic alkylating agents, including temozolomide (TMZ), in high-grade gliomas, particularly glioblastomas [8]. Since the protein is able to repair DNA lesions induced by alkylating factors, methylation of MGMT promoter (M-MGMT) results in better responsiveness to treatment with M-MGMT favoring the alkylating agents function; in fact, Everhard *et al.* found that M-MGMTP patients had a significantly longer progression-free survival after TMZ than U-MGMTP patients [9]. Our data confirm such a correlation, in that Patient 1 with M-MGMT responded to TMZ, while no response was observed in Patient 2 with U-MGMT.

## Conclusions

We report two cases of GC Type II carrying similar clinical and histological patterns but with distinct molecular and prognostic features. The poor prognosis of Patient 2 was underlined by molecular analysis which showed overexpression of the angiogenesis related genes *VCAM1* and *VEGF*, reflecting the high degree of neovascularization present. Moreover, in the same patient, we found a high expression of genes involved in drug resistance such as *ABCC3* (multidrug resistance protein 3, *MRP3*) and metallothioneins, which could play a role in intrinsic drug resistance, and, along with unmethylated MGMT status, may explain the lack of response to chemotherapy and overall poor prognosis of this patient. Current prognostic stratification of GC Type II is mainly based on tumor grade as assessed by the pathologist; our data, according to a few literature reports, seem to suggest that molecular characterization is required in order to provide proper subtyping and to identify targeted treatment strategies.
